# Controlled trial of a collaborative primary care team model for patients with diabetes and depression: Rationale and design for a comprehensive evaluation

**DOI:** 10.1186/1472-6963-12-258

**Published:** 2012-08-16

**Authors:** Jeffrey A Johnson, Fatima Al Sayah, Lisa Wozniak, Sandra Rees, Allison Soprovich, Constance L Chik, Pierre Chue, Peter Florence, Jennifer Jacquier, Pauline Lysak, Andrea Opgenorth, Wayne J Katon, Sumit R Majumdar

**Affiliations:** 1 Department of Public Health Sciences, University of Alberta, Edmonton, Canada; 2 ACHORD, University of Alberta, Edmonton, Canada; 3 Department of Medicine, University of Alberta, Edmonton, Canada; 4 Alberta Health Services, Edmonton, Canada; 5 Department of Psychiatry, University of Alberta, Edmonton, Canada; 6 Department of Psychiatry and Behavioral Sciences, University of Washington, Seattle, WA, USA

**Keywords:** Primary care, Collaborative, Diabetes, Depression, Controlled trial, Health services research

## Abstract

**Background:**

When depression accompanies diabetes, it complicates treatment, portends worse outcomes and increases health care costs. A collaborative care case-management model, previously tested in an urban managed care organization in the US, achieved significant reduction of depressive symptoms, improved diabetes disease control and patient-reported outcomes, and saved money. While impressive, these findings need to be replicated and extended to other healthcare settings. Our objective is to comprehensively evaluate a collaborative care model for comorbid depression and type 2 diabetes within a Canadian primary care setting.

**Methods/design:**

We initiated the TeamCare model in four Primary Care Networks in Northern Alberta. The intervention involves a nurse care manager guiding patient-centered care with family physicians and consultant physician specialists to monitor progress and develop tailored care plans. Patients eligible for the intervention will be identified using the Patient Health Questionnaire-9 as a screen for depressive symptoms. Care managers will then guide patients through three phases: 1) improving depressive symptoms, 2) improving blood glucose, blood pressure and cholesterol, and 3) improving lifestyle behaviors. We will employ the RE-AIM framework for a comprehensive and mixed-methods approach to our evaluation. Effectiveness will be assessed using a controlled “on-off” trial design, whereby eligible patients would be alternately enrolled in the TeamCare intervention or usual care on a monthly basis. All patients will be assessed at baseline, 6 and 12 months. Our primary analyses will be based on changes in two outcomes: depressive symptoms, and a multivariable, scaled marginal model for the combined outcome of global disease control (i.e., A1c, systolic blood pressure, LDL cholesterol). Our planned enrolment of 168 patients will provide greater than 80% power to observe clinically important improvements in all measured outcomes. Direct costing of all intervention components and measurement of all health care utilization using linked administrative databases will be used to determine the cost-effectiveness of the intervention relative to usual care.

**Discussion:**

Our comprehensive evaluation will generate evidence to reliability, effectiveness and sustainability of this collaborative care model for patients with chronic diseases and depression.

**Trials registration:**

Clintrials.gov Identifier: NCT01328639

## Background

Depression is one of the most common co morbidities in people with diabetes, present in 15-30% of patients with type 2 diabetes [1], at rates that are 30-40% higher than the general population [1,2]. Despite this, less than 50% of patients are recognized as having depression within the health care system [1,2]. Depression itself is a risk factor for the development of type 2 diabetes [3-5], and it is associated with adverse diabetes-related outcomes [6-9]. For example, comorbid depressive disorders in people with diabetes are associated with poorer self-care behaviors [10,11] worse glycemic control, higher risk of microvascular and microvascular diabetes-related complications [8-11], decreased quality of life and psychological well-being [2,12,13], and substantially higher health care costs [6,14,15]. The implications of untreated depression on long-term morbidity and mortality are even more significant when one considers that depression tends to be a recurrent condition, with 79% of diabetic patients with depression relapsing over a 5-year period with an average of 4 or more episodes [13,16]. In turn, diabetes may worsen the course of depression [4,16]. In fact, having one or more chronic medical condition, such as diabetes, increases the likelihood of developing a major depressive episode [17].

Although the majority of individuals with diabetes and depression are treated in primary care settings [18], only a minority of these individuals receive adequate treatment for depression [6,10,18]. Indeed, despite its high prevalence, less than one-half of all patients with depression are diagnosed or adequately treated [19,20]. Even primary care patients with established and treated depression receive only 48% of recommended management with both medication and psychotherapy [20]. It is also true that these rates of adequate treatment are much lower for those with diabetes, given that up to one-half of these patients have undiagnosed depression [1,19,20]. Depression has also been reported to be an important barrier to enhancing self-management, quality of care and outcomes in individuals with diabetes in primary care settings [18,21,22].

Traditional approaches to improving primary care of patients with complex comorbid chronic illnesses typically involves “carve-out” disease management programs, that is, delivering care for each condition, one disease at a time. Such single-disease management programs have been shown to be effective in improving control of conditions such as heart failure [23], diabetes [24,25], and depression [26]. However, a recent systematic review and meta-analysis of interventions for depressive disorders in patients with diabetes concluded that pharmacologic and collaborative care interventions were primarily aimed at and succeeded in the reduction of depressive symptoms, but, in general, had no effect on glycemic control [27]. It was suggested that individuals with comorbid physical and psychological problems require a far more integrated approach of care that targets such conditions collectively, and that management of these comorbid conditions should not be done separately [27].

This concept was recently proven in a pair of randomized trials investigating the efficacy of a case-management collaborative care model for patients with diabetes and depression in the primary care setting of Group Health Cooperative in Washington State [28,29]. In the first trial, a case-management approach proved effective in reducing depressive symptoms, but did not result in improved glycemic control [28]. In the second study, the case-managed collaborative care model was expanded in scope, aimed at achieving improvements of depressive symptoms and cardio-metabolic markers in individuals with depression and poorly controlled diabetes or heart disease [29]. In both cases, a nurse care manager guided patient-centered care with family physicians and consultant physician specialists. In the second study the intervention initially focused on the treatment of depression, but also aimed at management of diabetes and cardiovascular risk factors (e.g. high blood pressure, elevated lipids, elevated A1c), as well as lifestyle health-related behaviors. The trial involved 214 patients from 151 physicians in 14 primary care clinics. Patients in the intervention group had greater overall 12-month improvement across A1c (difference, 0.58%), LDL cholesterol (difference, 0.2 mmol/L), systolic blood pressure (difference, 5.1 mm Hg), and SCL-20 depression scores (difference, 0.40 points) (P < 0.001). Patients in the intervention group also were more likely to have one or more adjustments of antihypertensive medications (P < 0.001), insulin (P = 0.006), and antidepressant medications (P < 0.001), and they had better quality of life (P < 0.001) and greater satisfaction with care (P < 0.001). Patients in the intervention group had a mean of 10.0 in-person and 10.8 telephone visits with the nurse care manager over the 12-month period. The estimated mean cost per patient for this intervention, including all nurse contacts, specialist physician consultations, and information system support, was $1,224 (USD) in 2009 [29]. However, these intervention costs were offset by savings in total medical costs; over a 24-month period the intervention was associated with approximately $600 cost savings per patient compared to usual primary care [30].

While impressive, these findings need to be replicated and extended to other healthcare settings before the intervention model can be widely adopted or recommended. We took the previous randomized controlled trials as evidence of the efficacy of this case-managed collaborative model of care [28,29]. We therefore proposed a pragmatic approach in the implementation and evaluation of the collaborative care intervention in Primary Care Networks (TeamCare-PCN), and will employ a mixed-methods approach, based on the RE-AIM framework [31]. The RE-AIM framework examines five dimensions: Reach into target populations; Effectiveness; Adoption by target settings, institutions, and staff; Implementation, including consistency and cost of delivery; and Maintenance of effects in both individuals and settings over time. Thus, while a major focus of our protocol is on effectiveness, based on changes in clinical outcomes, applying the complete RE-AIM framework will allow us to determine the broader impact and transferability of the TEAM care model in the Alberta context.

## Methods

### Overall study design

**Figure 1:**
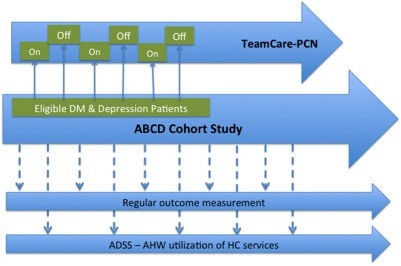
Schematic of Overall Design for TeamCare-PCN Study.

We will evaluate the effectiveness (i.e., the “E” in RE-AIM) of the TeamCare-PCN intervention using a controlled pragmatic trial design. We have implemented a variant of a practical design referred to as “cohort multiple RCT” (Figure 1) [32]. Patients with type 2 diabetes registered at PCNs will be invited to participate in an ongoing, annual survey as part of a larger Alberta’s Caring for Diabetes (ABCD) Cohort Study. Initial contact with cohort participants includes the administration of the Patient Health Questionnaire, a brief depression screening survey, and if positive for depressive symptoms, respondents will be invited to participate in the TeamCare-PCN study. We will allocate eligible participants to the intervention and usual care arms using an “on-off” time series (Figure 1); all participants will be assessed at baseline, 6 and 12 months.

**Figure 2:**
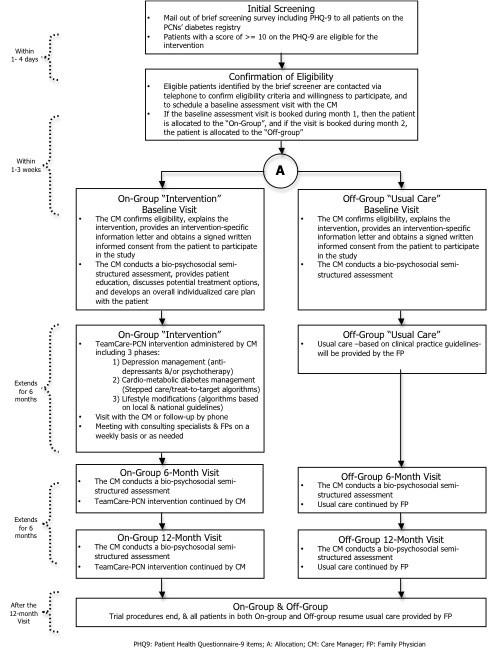
TeamCare-PCN Study Procedures.

Our decision to employ a pragmatic approach was based on a number of factors. First, the efficacy of the intervention has been established [28,29], so it is important that external validity now be accorded similar attention to the usual concerns related to internal validity [33-35]. Second, various forms of case-management have generally shown to be effective in improving quality of care for patients with diabetes [36]. Third, the effectiveness, applicability, and feasibility of the intervention have only been established in the urban managed care setting in the US [29]. Testing this intervention in a Canadian primary care setting is crucial to claim its effectiveness and applicability to the Canadian context. Finally, limitations in the available scientific information often impede the efforts of health policy makers and care providers in making evidence-informed policies for new and existing health technologies and interventions [33]. This results in allocating scarce healthcare dollars to interventions where the effectiveness and cost-effectiveness in routine clinical settings have not been evaluated [34,35,37]. While an economic evaluation of the original TEAM Care intervention was undertaken from the perspective of a US managed care organization provider [30], it is important to adopt a pragmatic approach to evaluate the effectiveness and cost-effectiveness of the TeamCare-PCN intervention in the Canadian context, and make this information available to support local health policy making and resource allocation.

### Hypotheses

We hypothesize the TeamCare-PCN intervention will reduce depressive symptoms, increase achievements of targets for cardio-metabolic measures, improve lifestyle behaviors, and be cost-effective. Furthermore, we anticipate this intervention will be acceptable and feasibly implemented in the PCN environment.

### Setting and population

TeamCare-PCN will be conducted in collaboration with four PCN in non-metro Alberta. A PCN is akin to the “medical home” concept [38], established as a network of primary care physicians in a given geographical area. Through the provincial Primary Care Initiative framework, funding is provided based on an approved business plan for the provision of primary care services to the local population. Each network has the flexibility to develop programs and to provide services in a way that works locally to meet the specific needs of patients. Funding may be used to establish central PCN office/clinic space, and hire allied health professionals, such as nurses, dieticians, pharmacists, or therapists. At the time of the launch of this study, there were 40 PCNs operating throughout Alberta, with about 80% of eligible family physicians working within a PCN. We elected to partner with PCNs outside of the larger metro centers because these cities already have greater access to specialist care as well as more established regional diabetes care and management programs than the non-metro PCNs. Collectively, the four participating study PCNs represent 140 general physicians and approximately 180,000 patients, with an estimated 10,000 patients with type 2 diabetes.

### Eligibility criteria

Inclusion Criteria:

 · Have type 2 diabetes and under the care of a PCN family physician and be

 · 18 years of age or older and

 · Score > =10 on the PHQ-9 [29,39,40] and

 · Speak English and have adequate hearing to complete telephone interviews and

 · Be willing and able to provide written informed consent to participate

Exclusion Criteria:

 · Severe and/or terminal physical illness (e.g. heart disease, renal failure, cancer, major organ failure) or

 · Serious and/or severe mental or psychiatric illness (e.g. bi-polar disorder or schizophrenia, use of anti-psychotic or mood stabilizer medication, or cared for by a psychiatrist); patients taking anti-depressants or receiving supportive psychotherapy from non-psychiatrists would be eligible or

 · Pregnant or breastfeeding or

 · Live in long-term care facility or

 · Already participating in other clinical trials

### Recruitment and allocation

Recruiting participants into TeamCare-PCN involves three steps. * First *, a screening survey accompanied with an endorsement letter from the PCNs will be mailed out to all patients with type 2 diabetes on the PCNs’ diabetes registries. This survey asks about age, the ability to read and speak English, having type 2 diabetes, length of time since diagnosis, and screens for depressive symptoms. * Second *, after potential participants have completed and returned the screening survey, PCN staff will contact eligible participants to confirm all eligibility criteria and if the participant is willing to participate, and schedule a baseline assessment visit. * Third *, during the baseline visit, the Care Manager (CM) will confirm eligibility, explain the intervention, provide an intervention-specific information letter and obtain a signed written informed consent from the patient to participate in the study.

Participants will be allocated to study groups using a previously successful “on-off” group assignment method (Figure 1) [39,41,42]. Eligible and consenting respondents who book for a baseline assessment with the CM during month 1 will be allocated to the intervention arm (ON-group). Those who book the baseline assessment in month 2 will be allocated to the usual care arm (OFF-group). This allocation process will continue until the target sample size is recruited. This method has been used in many quality improvement studies, reliably leads to balance in measured [and unmeasured] patient characteristics [39,41,42], and meets study design criteria for internal validity sufficient to permit inclusion in the Cochrane Collaborations’ Effective Practice and Organization of Care (EPOC) systematic reviews [40]. Because all four sites are involved in both “on” and “off” phases, this particular study design also balances case mix and controls for Hawthorne or volunteer effects, as well as issues related to “institutional learning” over time [39,40,42].

### TeamCare-PCN intervention

#### Overview

The TeamCare-PCN intervention involves a registered nurse Care Manager (CM), who coordinates collaborative team management for patients with diabetes and depression. Individuals with diabetes will be screened for depressive symptoms using the PHQ-9; patients with a score of > = 10 on the PHQ-9 are eligible for the intervention [43,44]. The PHQ-9 has been used in a number of interventions for screening of depressive symptoms in primary care [44], including among diabetic patients [45], and specifically in the efficacy study of the TEAM Care intervention [29].

The goal of the intervention is to reduce depressive symptoms, achieve targets for cardio-metabolic measures and improve lifestyle behaviors. The intervention includes three phases: (1) managing depression and improving depressive symptoms, (2) managing diabetes and controlling blood glucose, blood pressure and cholesterol, and (3) improving lifestyle behaviors such as healthy eating, physical activity and smoking cessation. The CM will coordinate the TeamCare-PCN intervention, while the family physician will remain responsible for final treatment decisions and all prescriptions. The CM will consult with specialists (psychiatrist and internist/endocrinologist) to consider management options depending on patients’ needs and desires. The CM will use locally endorsed evidence-based algorithms to provide recommendations for changes in the treatment plan, and in collaboration with specialists and the patients’ family physician, also support the implementation of the patients’ individualized care plan. Using a patient-centered focus, the CM will partner with the patient to develop a shared definition of problems, provide education and support, agree on specific targets/goals and individualized action plan, offer support and problem solving to optimize self-management, and closely monitor treatment adherence and outcomes.

#### Patient management

Patients entering the intervention will have a two-hour baseline appointment with the CM. This first visit will include a bio-psychosocial semi-structured assessment (reviewing medical history, previous treatments for depression and diabetes), patient education, potential treatment options (anti-depressant medications and/or psychotherapy) and developing an overall individualized care plan.

As mentioned, the intervention will consist of 3 phases; the first focusing on depression management, the second on cardio-metabolic diabetes management, and the third on general lifestyle modifications. A treat-to-target/stepped care approach will be used at each phase of the intervention. Recommended treatments have been based on algorithms that were developed in collaboration with the PCNs by compiling various guidelines and sources, such as extant clinical practice guidelines and consulting experts in the field. The CM will actively follow-up by telephone or in person one to two times per month to re-assess symptoms, and assist and support patients in achieving treatment goals. The CM will also have weekly meetings with the consulting specialists to review new cases and patient progress, and then communicate team treatment recommendations to the primary care physician.

The depression management phase (Phase 1) will involve the use of anti-depressant medications and/or referral for psychotherapy, as determined by the CM and the family physician (with study psychiatrist consultation as needed by the CM), tailored to each patient. A two-day training session for CM and consultant specialists was planned at the beginning of the project, with an annual one-day booster session The CM will be given basic training in three psychotherapeutic techniques during this phase: problem-solving therapy [46-49], behavioral activation [50-54], and motivational interviewing [55-57]. Direct referral to the care of a psychiatrist will occur only on failure of two separate trials of anti-depressants, or one trial of anti-depressant in combination with psychotherapy. Once patients have reached remission, the CM and patient work together towards a relapse prevention plan to help the patient identify when and where to seek help with future depressive symptoms or renewed problems for disease control. The CM will continue to work with patient through Phases 2 and 3 of the intervention.

Stepped care/treat-to-target algorithms for Phase 2 (cardio-metabolic care) were developed in collaboration with health care professionals at the PCNs and study specialists using local and national guidelines. Phase 2 includes working with the CM in analogous fashion to Phase 1, but with a focus on reaching individualized targets for hemoglobin A1c, lipids and blood pressure measures. Phase 3 involves patient education to address lifestyle behaviors such as diet and exercise. Locally developed educational materials and existing PCN support programs will be available for patient referrals.

### Usual care

All participants in TeamCare-PCN will be actively screened for depression and those allocated to usual care (or intervention) will be identified as such to their family physician. For disease management, patients will receive care from their family physician, without additional active support from the CM. Therefore, this group will receive the usual standard care for diabetes and depression from their family physicians based on available clinical practice guidelines. Participants in this group will be assessed for all of the same outcome measurements as intervention patients. A recently published trial concluded that screening for depression with written feedback to patients and physicians resulted in no improvements in depressive symptoms, nor changes in utilization of mental health care services [58]. Thus, in our design, the usual care group is very likely to represent usual care for patients with diabetes and depression.

### Outcome measures

To assess the effectiveness (“E” in RE-AIM) of TeamCare-PCN, we will use two primary outcome measures. First, given that we selected patients on the basis of depressive symptoms, our primary depression-related outcome will be the PHQ-9 scores at 12 months. Second, a global measure of patient-centered improvement (the scaled marginal model, see Analysis below) based on the combination of chronic disease-related clinical outcomes. All outcomes will be measured in all participants (i.e., both groups) at baseline, 6, and 12 months. We will secondarily evaluate process indicators for care received, as well as a host of patient-reported outcome measures to determine the overall effectiveness of the TeamCare-PCN intervention.

#### Depression-related outcome

The outcome measure for depression will be improvement in PHQ-9 score [43,44]. The minimal important difference on the PHQ-9 score is widely considered to be 5 points [44]. A remission of depression symptoms is indicated with a score <10 for a period of three consecutive months [29]. Operationally, this can be conceptualized as a continuous score or as a binary outcome: improved vs not, since all participants will have scores greater than 10 as part of their eligibility.

#### Chronic disease-related clinical outcomes

Clinical outcome measures include systolic blood pressure (SBP), LDL cholesterol and A1c, demonstrated by achievement of targets or significant improvements as indicated with a 10% improvement over baseline [59,60]. These outcomes will be measured using point-of-care devices that we have standardized and placed in each PCN. Capillary blood samples will be collected from participants to assess A1c and lipid profile. Cardiovascular (blood pressure and resting heart rate) and anthropometric (weight, height, and waist circumference) measurements will also be assessed at clinic visits. Regular, monthly quality assurance checks will be conducted on the point-of-care devices, validated against central laboratory measurements.

#### Process of care indicators

Process indicators will include the number of visits with PCN care providers, including CM, family physicians, specialists consults, referrals for mental health care, and use of medications and psychotherapeutic sessions. The time spent with patients will be tracked for all CM contacts. Adjustments to medications and adherence to treatments will also be assessed.

#### Patient-reported outcomes

A range of patient-reported outcomes will be collected from all participants by regularly administering previously validated surveys at baseline, 6 and 12 months. Survey sections and measures are outlined below, and a briefer version of the survey will be used at 6-months. These measures of patient-reported outcomes were selected based on extensive literature supporting their psychometric properties and common application in assessments of this patient population

 · *Health Related Quality of Life:* includes assessment of general health status (SF-12 and EQ-5D) [61-64] and diabetes-specific stress (Problem Areas in Diabetes 5-item) [65].

 · *Health behaviors and self-management:* includes assessment of smoking behaviors, alcohol consumption [66], substance use [67], and physical activity [68], and the Summary of Diabetes Self Care Activities (SDSCA) [69].

 · *Satisfaction with care*: using Consumer Assessment of Healthcare Providers and Systems (CAHPS), Adult Primary Care 1.0 [70] and Patient Assessment of Chronic Illness Care (PACIC) [71,72].

 · *Health Literacy and Self-efficacy:* includes assessment of health literacy (3-brief screening questions [73] and self efficacy (Stanford Self-Efficacy for Managing Chronic Disease 6-Item Scale) [74].

### Data management

The depression and medical outcome measures will be entered into a clinical management tracking system as part of the TeamCare-PCN intervention. The data collected in the survey and through clinical measurement will be entered into centralized, web-accessible databases. These study databases will be housed on secure servers in the research offices at the University of Alberta. Double data entry will be conducted and research staff will remain masked to allocation status at all times. Once the study is completed, all data will be exported and merged, based on individually assigned study ID numbers, to form an analyzable dataset. Investigators, research assistants, and analysts will be masked to allocation status at all times.

### Data analysis

As the initial focus of the intervention is on the management of depressive symptoms, we have considered the PHQ-9 as a main primary outcome, and will assess changes in PHQ-9 scores over 12-months between groups, adjusting for baseline PHQ-9 score, using a mixed effects multivariate model. For our second primary outcome of improvements in global disease control, we will employ a multivariate model that jointly tests the changes in multiple clinical outcomes, namely A1c, LDL cholesterol and systolic blood pressure [29]. Using the same analytic approach as in the recently published RCT of this intervention, we will apply a scaled marginal model [29,75] to simultaneously compare the change in these continuous outcomes at 12 months, adjusting for the baseline status for each variable. This approach scales the changes in each outcome by its standard errors, and thus the coefficients can be directly interpreted as effect sizes [29]. In either primary outcome analyses, the models will be estimated iteratively and the potential correlations among and between outcomes within individuals will be handled using generalized-estimating equation (GEE) models for each outcome [75].

We will also compare the study arms based on the proportion of subjects who achieved a remission of depression (i.e., PHQ-9 < 10 for 3 consecutive months) and the proportion of patients who achieve a clinically important change in the outcomes (that is, achieving a reduction of 5 points on the PHQ-9 [44] or a 10% or greater improvement over baseline values for A1C, LDL cholesterol and SBP at 12 months) [59,60], using generalized estimating equations and multivariable logistic regression models. This approach will allow us to present and interpret all outcomes in terms of absolute differences and a number-needed-to-treat [76].

For all analyses we will employ an intention-to-treat framework for our primary analysis, using a last-observation-carried-forward method of imputation for subjects who do not have complete follow-up data for primary or secondary outcomes. This approach is conservative as it assumes subjects with missing follow-up data have not changed. In either case, the regression models will accommodate additional covariates, as required, to adjust for PCN site, as well as any post-allocation baseline differences between groups in clinically important (e.g., age, sex) or statistically significant (e.g., p < 0.1) characteristics. This ability to statistically control for potential imbalance may be necessary given the non-random treatment allocation used in our “on-off” design.

### Sample size and power considerations

We estimate that a minimum total sample size of 120, with 60 in each arm, would provide power of 0.80 to detect a mean difference of 5 points in the PHQ-9 [44], assuming a repeated measures correlation of 0.6, and 2-tailed alpha of 0.05. This sample size also provides more than 80% power (two sided alpha = 0.05) to detect any between-group absolute difference in proportions of 15% or more (e.g., 45% of usual care patients achieve depression remission vs 60% of intervention patients) [29]. Anticipating a 40% attrition rate, we planned to recruit 168 patients in total across our 4 PCN sites. Although the model is implemented in 4 PCN sites, the individual general physicians affiliated with the PCN will carry out the changes in care. While we will include PCN site as a covariate in our analysis, because there are approximately 140 general physicians across these 4 PCN sites, we choose not to further inflate our sample to account for clustering of patients and their outcomes.

### Broader RE-AIM evaluation

#### Reach

Using demographic information and eligibility criteria, a comparison of patient characteristics between the intervention and usual care groups will be conducted to address reach. Aggregate demographic information between participants and non-participants will be compared using population-level data available through the Alberta Diabetes Surveillance System [77]. Information on reach will also be documented through a patient recruitment tracking system, the PCNs’ diabetes patient registries, and monthly reports on recruitment submitted by PCN staff.

#### Implementation, including cost-effectiveness

Implementation evaluation is used to examine the extent to which an intervention is delivered as planned, particularly in real world practice settings. This type of evaluation explores why an intervention is successful or not, and can contribute to the successful realization of programs in the future. Implementation evaluation of TeamCare-PCN will enhance our contextual understanding and interpretation of why the intervention had an impact or not, in which settings (i.e. PCNs), and for whom. It also provides a mechanism for continuous quality improvement by generating ongoing information. Implementation evaluation is also crucial to determine the sustainability of a program, as it is used to inform future decision-making and strategic planning by identifying critical successful factors for implementation (such as resources, staff qualities, and leadership) and recommendations to mitigate barriers.

We will examine the intended versus actual implementation of TeamCare-PCN through several means: document review; pre/post training survey of CMs to assess knowledge and confidence with the model; usual care checklist documenting organizational priorities and systems to implement the model; baseline, mid- and post-intervention interviews with PCN staff regarding facilitators and barriers to implementation and recommendations for improvement; and participant-observation.

A number of economic evaluations of team-based interventions in patients with diabetes and depression have been conducted [30,78-80]. Incremental cost-utility ratios have been estimated at less than $400 per quality-adjusted life year gained [78]. While the consistent economic benefit of these interventions has been demonstrated, these evaluations are highly dependent on many factors including the implementation strategies of these interventions in the primary care settings of the single urban managed care environment in the US. We will therefore conduct an economic evaluation of the TeamCare-PCN implementation, using a similar methodological approach. To do this, we will use primary data collected during the study period, health care utilization data obtained through linkages with administrative databases, as well as an Alberta-based economic model to estimate future diabetes related costs [81]. For our economic evaluation models, we will take the perspective of the individual PCN Board as the main decision maker, with a second perspective of the third-party payer, that is, the provincial health care system, along with our analyses focusing on direct medical costs, as this is the most relevant perspective for our decision-making partners.

##### Intervention costs versus usual care costs

Costs for the intervention will be estimated using the actual salary and benefits for the Care Manager and Administrative Assistant provided for the intervention plus a 30% overhead rate for PCN space and management costs [82]. The CM time will be estimated based on the actual visits with intervention patients, for both in-person and telephone contacts, as well as outreach efforts and record-keeping. Intervention costs will also include a fixed amount for each participant assigned to the intervention program to defray the costs of specialist supervision and the clinical management support.

##### Health care utilization data

Patients enrolled in the study will be asked for permission to access their medical records by providing their personal health number, thus allowing linkage to provincial health care administrative data from Alberta Health and Wellness (AHW) for physician, hospital, and emergency department billing, and pharmaceutical data (for patients 65 years and older). This linkage will allow health care utilization and health care costs to be included in the evaluation. These data sources are regularly monitored as part of the Alberta Diabetes Surveillance System (ADSS) [77].

##### Long-term economic projection model

We have developed a projection model for the future burden of diabetes in Alberta using data collected for the ADSS [81]. Our projection model is based on two elements: epidemiologic trends and costs. To estimate the future incidence of diabetes complications, we will use trends in incidence rates by diabetes status, sex and age group. We will model the longer-term (e.g., at 10-, 20- and 30-years) costs and outcomes of the TeamCare-PCN intervention. We will employ a discount factor of 5% to all future costs and benefits, and perform sensitivity analyses on this discount factor, using rates of 0%, 3% and 10%. Last, we plan to conduct exhaustive one-way, multi-way, and probabilistic sensitivity analyses to ensure our findings are robust [82,83].

#### Adoption

We will examine the adoption of TeamCare-PCN by documenting and comparing the characteristics of the participating PCNs. This will be done through document review (e.g., PCN websites, business plans), the usual care checklist (description of participating PCNs - including number of family physicians, number of patients, governance structure, organizational processes and structures), and interviews with PCN staff. Determining the representativeness of the participating PCNs compared to non-participating PCNs represents a challenge and will be dependent on the availability of secondary data to describe the characteristics of all PCNs in Alberta.

#### Maintenance

We will also examine the extent to which the effects of the interventions are sustained over time in patients (that is, sustained awareness, knowledge, and management of T2D, depression, and lifestyle behaviors) and at the organizational level of the PCN. This type of information will be gathered through patient reported outcomes (e.g., health behaviors and self-care items) through regular surveys at baseline, 6 and 12 months. These survey items will be collected through the ABCD Cohort Study survey, which will be administered annually for five years after completion of TeamCare-PCN. Also, interviews with PCN staff will be conducted regarding decisions around incorporating the intervention models into future business planning or continuing the use of aspects of the model, such as using the diabetes registry to inform PCN programming, or using screening tools to identify and assess depressive symptoms among people with chronic illness.

### RE-AIM data and analysis

The primary data sources for our broader RE-AIM evaluation activities include the following:

 1) Project documents review including patient tracking databases, email communications, and review of secondary data, as available;

 2) Participant observation includes assessment of notes generated from meetings with the PCNs and other partners; and,

 3) Interviews with the Executive Directors, Chronic Disease Clinical Managers, and the CMs from the 4 participating PCNs. Specialists and family physicians will also be invited to participate.

Interviews will be digitally recorded, transcribed verbatim by an independent transcriptionist and verified for accuracy. All qualitative data sources will be compiled and managed using NVivo 9.0 software. We will take a general inductive approach to analysis of the qualitative data [84], which places fewer restrictions on the content analysis than more constraining approaches such as grounded theory or phenomenology [85]. Within this approach, the evaluation questions related to the RE-AIM framework will direct analysis of the data. However, the findings will be derived directly through a content analysis [86].

### Ethics and funding considerations

All patient participants will receive information about the study and the opportunity to ask any questions. Written informed consent will be obtained from participants prior to obtaining any study measurements. Ethics approval for the study and its evaluation has already been granted from the Health Research Ethics Board (HREB #PRO00012663) at the University of Alberta. The broader evaluation component of this study was also submitted to the HREB for review. However, the Board deemed this component of the study as evaluation and not research; it was deemed to not require ethics review and approval. Regardless, the requirements outlined in the Tri-Council Policy Statement: Ethical Conduct of Research Involving Humans [87] will be followed in this study. Verbal informed consent from participants will be sought prior to any evaluation activities.

The funding for this study is from a contract from the provincial government (Alberta Health and Wellness) to the Alliance for Canadian Health Outcomes Research for Diabetes (ACHORD) Group. The funding source had no role in the design of the study and will have no role in the conduct, analysis or reporting of the study, nor access to the data.

## Discussion

We report the protocol for the design and comprehensive evaluation of a collaborative team model of primary care for patients with comorbid depression and diabetes. Diabetes is a common and increasingly prevalent chronic medical condition. Comorbid depression is common in people living with diabetes, although it often goes unrecognized and therefore untreated. Together, diabetes and depression increase the risk of adverse health outcomes in these patients, including increased mortality and increased heath care utilization, and treatment should be administered using an integrated approach that targets both diabetes and depression collectively. The efficacy of this collaborative care model has been demonstrated, but only within one managed care environment in the US. Our proposal is to evaluate this model of care in the evolving primary care environment in Canada.

We have proposed a pragmatic approach in the study design of the TeamCare-PCN intervention, and will employ a mixed methods approach for our comprehensive evaluation. Therefore, while we will undertake a controlled *outcome evaluation* based on changes in clinical parameters to determine effectiveness, we have also planned an *economic evaluation* and qualitative assessments of reach, adoption, implementation and maintenance. This broad evaluation is intended to provide the relevant decision-makers (i.e., provincial funding agencies and primary care networks) with stronger information on which to build business plans for future service delivery. Ultimately, we believe our work will serve as a platform upon which an emerging model of primary care can incorporate an effective and cost-effective depression intervention into the management of individuals with type 2 diabetes, and as a framework for implementing and evaluating similar interventions for other chronic conditions.

## Competing interests

Dr. Chue has received research and travel grants from Janssen, Pfizer, Astra Zeneca, Lund beck, Bristol Myers Squibb and Eli Lilly as a researcher and speaker.

Dr. Katon has received support to serve on advisory boards from Eli Lilly and has received honorariums for lectures from Pfizer, Forest, and Eli Lilly.

The remaining authors declare that they have no competing interests.

## Authors’ contributions

JAJ and SRM conceived and designed the study, based on a previous study led by WJK. WJK served as a consultant in the implementation phase of this study. JAJ and FAS drafted this manuscript, with all authors providing critical comments and revisions. All authors have read and approved the final version.

## Pre-publication history

The pre-publication history for this paper can be accessed here:

http://www.biomedcentral.com/1472-6963/12/258/prepub
